# Prevalence of TTI among Indian blood donors

**DOI:** 10.6026/97320630019582

**Published:** 2023-05-31

**Authors:** Sanjay Kumar Thakur, Sompal Singh, Dinesh Kumar Negi, Anil Kumar Sinha

**Affiliations:** 1 P.G. Department of Zoology, Veer Kunwar Singh University, Ara, Bihar, India - 802301; 2 Department of Regional Blood Transfusion Centre, Hindu Rao Hospital and NDMC Medical College, Delhi, India – 110007

**Keywords:** Blood Donors, Transfusion Transmissible Infections, Blood group

## Abstract

Transfusion Transmissible Infections (TTIs) such as human immune-deficiency virus (HIV-I/II), hepatitis B virus (HBV), Hepatitis C virus (HCV), Malaria parasite (MP) and syphilis can spread through contaminated blood or blood products. The present study was designed to analyze the prevalence of TTIs and their association with blood group, among the blood donors of Delhi. Blood group was determined by hem-agglutination using Gel card. HIV, HBV, and HCV test was performed by ELISA, syphilis by RPR and MP rapid card method. A total Transfusion Transmissible Infections (TTIs) such as human immune-deficiency virus (HIV-I/II), hepatitis B virus (HBV), Hepatitis C virus (HCV), Malaria parasite (MP) and syphilis can spread through contaminated blood or blood products. The present study was designed to analyze the prevalence of TTIs and their association with blood group, among the blood donors of Delhi. Blood group was determined by hem-agglutination using Gel card. HIV, HBV, and HCV test was performed by ELISA, syphilis by RPR and MP rapid card method. A total of 345(2.038%) blood donors were positive for TTIs. Prevalence of HBV, HCV, HIV-I/II, syphilis and MP were 188(1.111%), 73(0.431%), 34(0.201%), 49(0.29%) and 1(0.006%) respectively. Our result shows a trend of decrease in prevalence of TTIs; 2.267%, 2.111% and 1.614% between the year 2020, 2021 and 2022 respectively. Significant association of syphilis infection (P=0.036) and HCV infection (P=0.012) with ABO blood group antigen was observed. Blood group O donors were 1.81 times more infected with syphilis compared to donor having A and B antigen. Donors having blood group antigen B were 1.80 times more infected with HCV compared to donor not having B antigen. HBV and HIV prevalence found to be not associated with ABO and Rh blood group antigens. A low prevalence of TTIs positivity was observed among blood donors. Public awareness, proper counseling, medical examination and testing can help to minimize TTIs. Our study results shows ABO blood group has an association with HCV and VDRL infection.

## Background:

Transfusion Transmissible Infections (TTIs) are infections that can spread from one person to another through the use of contaminated blood or blood products. The term "blood transfusion infections" refers to a range of illnesses, the most prevalent of which are hepatitis B virus (HBV), human immunodeficiency virus (HIV-I/II), hepatitis (HCV), and syphilis. In India it mandatory to test TTIs before blood transfusion includes HBV, HCV, HIV-I/II, malaria parasite (MP) and syphilis or VDRL (venereal disease research laboratory). In addition to the significance of blood groups in blood transfusion, different blood groups are associated with different human pathogens and especially with TTIs, it poses health risks and plays a significant role in blood transfusion [1, 2].

Blood group antigens serve as a representation of people's polymorphic characteristics. Karl Landsteiner discovered the first human ABO blood group system in 1901. Later, in 1941, Landsteiner and Wiener defined the Rh system [3, 4]. Individuals are classified into the four main ABO blood groups-A, B, O and AB, based on the presence of antigens (agglutinogens) on the surface of red blood cells and corresponding antibodies (agglutinins) in their plasma. Presence of Rh(D) antigen determine positive and its absence determine Rh negative blood group in the Rh system. The H antigen is created by the addition of 1-2 fucose by FUT1 or H-glycosyltransferase. H antigen can serve as a substrate for ABO glycosyltransferase. Those having blood group A express 1-3 N-acetylgalactosamine (GalNAc) (Gal), While those having blood group B express 1-3 galactose. On the other hand, people in Group O only express the H-antigen precursor and have dormant ABO genes [5]. Microbes and environmental material that resembles A/B-antigens have been shown to trigger naturally occurring ABO system antibodies against the antigens of the ABO blood group [6]. The body's innate immune system uses ABO antibodies to attack dangerous bacteria and viruses that have ABO-active antigens. The innate immune response to an infection can also vary depending on blood type [5, 7]. On the other hand, blood groups have the capacity to serve as pseudo receptors. Certain blood groups are used as receptors and legends by bacteria, viruses, and parasites. For instance, Plasmodium vivax and other malarial parasites can bind to the Duffy blood group antigen [8, 9]. Additionally, some blood types' antigens facilitate membrane micro-domain retention, cell adsorption, and/or signal transmission. Research suggests that TTI agents can't attach to the polysaccharide if ABO antigens are present. On the other hand, cells lacking these antigens run the risk of acquiring TTIs [5,10]. The host's susceptibility to certain infections may be increased or decreased by variations in blood group antigen expression [11]. Recently a study on COVID-19 infection, shows persons having blood group A and B were more susceptible to COVID 19 infection while persons having blood group O were less susceptible [12].

The three most common viruses that cause death worldwide are hepatitis C virus (HCV), hepatitis B virus (HBV), and HIV [13]. According to statistics, there are 71.0 million people who are HCV positive, 25.70 million people who are HBV positive, and 36.70 million people who are HIV positive worldwide. According to estimates, 2.30 million and 2.70 million patients, respectively, had co-infections with HIV/HCV and HIV/HBV as a result of the same method of transmission [14]. The life expectancy at birth increased by 2.9 years, for men and 2.6 years for women in Tuscany, Italy. Male and female life expectancy decreased by 0.11 and 0.16 years, respectively, due to an increase in infectious disease mortality [15]. Hepatitis B and C prevalence rates were 6.7% and 14.3%, respectively, according to a study carried out in Pakistan, with co-morbidities of HIV/HCV at 80% and HBV/HCV at 20% [16, 17]. A chronic hepatitis B infection increases a person's risk for cancer, liver cirrhosis, and a number of other diseases. This illness is highly contagious [18]. HCV and HBV infections are well-known among people living with HIV due to their comparable viral transmission routes. Co-morbidities, like liver problems brought on by HCV or HBV infection, are a significant concern in HIV-infected individuals [19]. Therefore, it is of interest to analyze the prevalence of TTIs and their association with blood group, among the blood donors of Delhi.

## Methods:

## Ethical Considerations:

Present study was approved by the institutional ethical review committee of Hindu Rao Hospital and NDMC Medical College, Delhi by the approval number- F.No: IEC/NDMC/2021/69. The blood donation consent was obtained from all the participant blood donors. For present study, only data of routine blood grouping and TTIs screening test results of blood donors from blood bank inventory registers were used. For present study no separate blood sample was obtained from donors, hence the separate informed consent was not obtained.

## Blood donation:

All biosafety precautions were taken and infection control protocols were followed, during the procedure for collecting and testing of blood samples. All volunteer and replacement blood donors who arrive at the blood bank undergo counseling and evaluation before donating blood as per standard operating procedure (SOP).

## Inclusion criteria:

Only those blood donors who are healthy and have no risk of developing TTIs are chosen for blood donation. Donors had not a recent, past or present history of hepatitis, some chronic diseases, sexually transmitted diseases, surgery, asthma, high-risk activity (like random unprotected intercourse), and pregnancy. The study comprised blood donors who were in good physical health, were between the ages of 18 to 65 year, weight >45 kg and hemoglobin levels >12.5 gm/dl.

## Exclusion criteria:

Donors not qualified for blood donation were excluded from this study.

## Serological testing:

The ABO and Rh blood grouping was performed by hem-agglutination test using Gel card method (DiaClon ABO/D+Reverse Grouping, BIO-RAD, Switzerland) according to manufacturer instructions. The presence of A and B antigens on the surface of red blood cells (RBCs) and corresponding antibodies, anti-A or anti-B antibodies, in the serum allowed for the identification of the ABO blood types. As opposed to blood group B, which contains antigen B on the RBC surface and anti-A antibody in the serum, blood group A has antigen A on the surface of the RBC. The blood group type AB, however, possesses both A and B antigens on the surface of RBCs but does not have anti-A or anti-B antibodies in serum. Although the serum contains both anti-A and anti-B antibodies, the RBC surface of blood group type O is devoid of any antigens.

Donor's blood was screened for TTIs after donation. Tests were carried out using commercially available kits in accordance with the manufacturer's instructions. The blood samples were tested for HIV 1&2, HBsAg and HCV by standard enzyme-linked immunosorbent assay (ELISA) test kits. The hepatitis B surface antigen (HBsAg) was detected using MonolisaTMHBs Ag ULTRA (BIO-RAD, Marnes-la-Coquette - France), and the kit had a sensitivity of 100% and specificity of 99.94%. The combined screening for Anti-HCV antibodies and viral antigen of the hepatitis C virus in serum/plasma were performed by using MonolisaTMHCV Ag-Ab ULTRA V2 (BIO-RAD, Marnes-la-Coquette - France) which had a sensitivity of 100% and specificity of 99.94%. Screening test for the detection of HIV P24 Antigen and antibodies to HIV-1 and HIV-2 in human serum/plasma were performed by using GenscreenTMULTRA HIV Ag-Ab (BIO-RAD, Marnes-la-Coquette - France). The kit had a sensitivity of 100 % and specificity of 99.95%. Antibody for treponemapallidum was tested using rapid plasma reagins(RPR) carbon antigen test (RECKON DIAGNOSTICS P. LTD., Gorwa, Vadodara, India). Screening test for Malaria parasite antigen, Plasmodium falciparum (Pf) and Plasmodium vivax (Pv) in human blood were performed by using Malaria Pf/Pv Ag Rapid test a lateral flow chromatographic immunoassay (BIOGENIX Inc. Pvt. Ltd. Lucknow, India).

## Statistical analysis:

Study data were collected from blood bank inventory registers and entered into Microsoft Excel spreadsheets. Data analysis was performed using open source statistical software R version 4.0.0 (R, USA). The prevalence of HIV, HCV, HBV, MP and syphilis were expressed in percentages. The difference in between male and female donor's hemoglobin level, age and weight were analyzed using students t-test. Association between the blood groups and TTIs were done using Pearson Chi-Square test. The associations are presented as odds ratio (OR) together with 95% confidence intervals (CI). P-value less than 0.05 were considered statistically significant.

## Result

Of a total of 16925 blood donors, 16777 (99.12%) were male and 148 (0.87%) were female. The male donor's age was 31.16 ± 8.43 years and female donor's age was 33.33 ± 9.50 years. The difference in age of male and female donors was not statistically significant (p=0.108). The male donor's hemoglobin level was 14.58±1.58 gm/dl and female donor's hemoglobin level was 13.44±0.85 gm/dl. The difference in hemoglobin level of male and female donors was statistically significant (p=0.00). The male donor's weight was 74.29±11.73 Kg and female donor's weight was 65.54±8.01 Kg. The difference in weight of male and female donors was statistically significant (p=0.00).

## Prevalence of Transfusion-Transmissible Infections among studied blood donors:

Donor's blood tested for blood group and TTIs. The results are given in (Table 1). A total of 345 (2.038%) blood donors were positive for TTIs, in which A, B, O, AB, Rh positive and and Rh Negative were 72 (0.425%), 145 (0.857%), 97 (0.573%), 31 (0.183%), 328 (1.938%) and 15(0.089%) respectively. Among these, only one blood donor having blood group "O" Rh positive was tested positive for MP. Of a total of 148 female donors only one donor having blood group A Rh (D) positive was screened positive for HIV-I/II.

**Figure 1:**
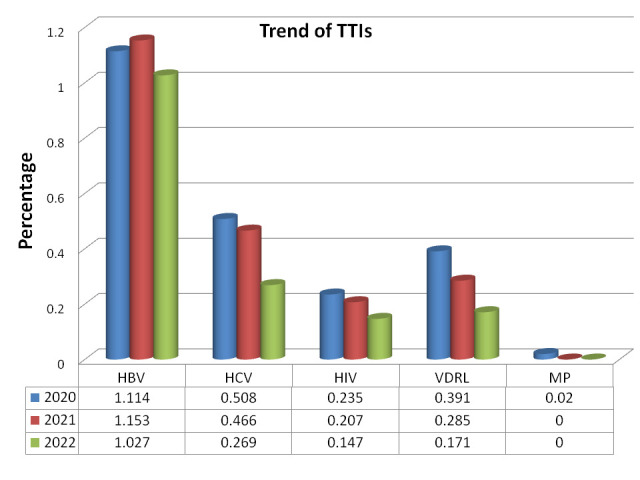
year wise trend of TTIs in year 2020, 2021 and 2022.

Present study result shows a steady trend of decrease in cumulative frequency of TTIs positivity between the year 2020, 2021 and 2022, were 2.267%, 2.111% and 1.614 respectively. Among these, only one blood donor in year 2020 having blood group O Rh (D) positive was tested positive for MP. Among TTIs, only HBV shows an increased frequency in year 2021 compared to year 2020 that decreases in year 2022 (Table 2, Figure 1).

A total of 188(1.111%) blood donors were positive for HBV, in which A, B, O, AB, Rh positive and Rh Negative were 44(0.26%), 76(0.449%), 51(0.301%), 17(0.1%), 180(1.064%) and 8(0.047%) respectively. A total of 73(0.431%) blood donors were positive for HCV, in which A, B, O, AB, Rh positive and Rh Negative were 12(0.071%), 39(0.23%), 13(0.077%), 9(0.053%), 67(0.396%) and 4(0.024%) respectively. A total of 34(0.201%) blood donors were positive for HIV-I/II, in which A, B, O, AB, Rh positive and Rh Negative were 6(0.035%), 14(0.083%), 12(0.071%), 2(0.012%), 33(0.195%) and 1(0.006%) respectively. A total of 49(0.29%) blood donors were positive for VDRL, in which A, B, O, AB, Rh positive and Rh Negative were 10(0.059%), 16(0.095%), 20(0.118%), 3(0.018%), 47(0.278%) and 2(0.012%) respectively.

There were 5 donor tested positive for both HCV and HIV-I/II, one having blood group A Rh(D) negative, one having A Rh(D) positive, two having blood group B Rh(D) positive and one having blood group AB Rh(D) positive. There were 2 donors tested positive for both HBV and HCV, one having blood group A Rh(D) negative and one having B Rh(D) positive. There was 2 donor tested positive for both HIV-I/II and VDRL, one having blood group AB Rh(D) positive and one having O Rh(D) positive. There was one donor having blood group B Rh(D) positive for both HCV and VDRL.

## Association of Blood group with TTIs:

The genes responsible for the synthesis of ABH and Rh antigens are located on different chromosomes. Thus it is not fair to consider the "ABO and Rh(D)" phenotype as a single and homogeneous entity, considering this fact, in the present study ABO and Rh (D) blood group system analyzed separately to obtain reliable results.

## HCV:

The comparative percentage frequency of HCV positive was higher (Table 3) in donors having blood group antigen B and lower in donors having blood group A and O (not having antigen B). In HCV positive donors, a significant association (χ²=6.279, P=0.012) between donors having blood group B antigen (B+AB) and not having B antigen (A+O) was observed. Donors having blood group antigen B (B+AB) were more infected with HCV (OR=1.8098, CI=1.1303 to 2.8980, z statistics=2.470, p=0.0135) compare to non B antigen blood group (A+O) having less infection ratio (OR=0.5525, CI=0.3451 to 0.8847, z statistics=2.470, P=0.0135). Although in Rh(D) negative donors, HCV positive was higher (9.46%) and negative was lower (4.96%) compared Rh(D) positive donors having HCV negative higher(94.45%) and positive lower (90.54%), the association was not statistically significant (χ² =3.091; p=0.079).

## VDRL:

The comparative percentage frequency of VDRL positive was higher (Table 3) in donors having blood group O (not having antigen A and B) and lower in donors having blood group antigen A and B. In VDRL positive donors, a significant association (χ²=4.382, P=0.036) between O blood group donors (not having antigen A and B) and donor having A and B antigen (A+B+AB) was observed. Donors having blood group O (without antigen A and B) were more infected with VDRL (OR=1.8158, CI=1.0302 to 3.2004, z statistics=2.063, p=0.0391) compare to donors having A, B and AB antigen (A+B+AB) which have less infection ratio (OR=0.5507, CI=0.3125 to 0.9707, z statistics=2.063, P=0.0391). The Rh(D) positive and negative blood group of VDRL positive and negative blood donors shows a similar pattern (Table 3) and association was not statistically significant (p=0.765).

## HBV:

The percentage frequency of ABO and Rh blood group of HBV positive and negative blood donors (Table 3) shows a similar pattern with slight variation (Table 4). The association between HBV infection and ABO and Rh blood group antigen was not statistically significant (p>0.05).

## HIV-I/II:

The percentage frequency of ABO blood group of HIV-I/II positive and negative blood donors (Table 3) shows a slight variation. The comparative frequency of HIV-I/II positive was higher in B and O blood group and lower in A and AB (Table 4). However, association between HIV-I/II infection and ABO blood group antigen was not statistically significant (p>0.05). Although in Rh(D) positive donors HIV-I/II positive was higher (97.06%) and negative was lower (94.43%) compared to Rh(D) negative donors having HIV-I/II negative higher(4.98%) and positive lower (2.94%), the association was not statistically significant (χ² =0.306; p=0.58)

## Discussion:

The demographic pattern of our blood donors showed 99.12% were male and only 0.87% were female. The mean of male donor's hemoglobin level was significantly higher (14.58 ±1.58 gm/dl) than the female donor's (13.44±0.85 gm/dl). The mean weight of male donor's was also significantly higher (74.29±11.73 Kg) than the female donor's (65.54±8.01 Kg). Likewise, the study conducted in the Coastal South India 95.2% of blood donors were males [20]. Almost similar findings has also been from Brazil (99.6%), Western Region of Saudi Arabia (96.9%), Central Region of Saudi Arabia (82.98%), Ethiopia (86.8%), Cameroon (82.0%) and Nigeria (81.9%) [20]. This may be attributable to the cultural stigma in some places that states that, women shouldn't donate blood because they already lose blood on a regular basis from menstruation and that doing so could weaken them and put their health at risk. Other research from various contexts, however, revealed that the proportion of male and female blood donors was roughly equal.. For instance, the Belgium (54.6%), Spain (54.0%), United States (51.7%), Netherlands (50.0%),the France (50.0%), Denmark (50.0%), United Kingdom (47.0%) and Finland (45.0%) [20].

Our study results shows, overall cumulative frequency of TTIs in blood donors was 2.038%, and frequency of HBV, HCV, HIV-I/II, VDRL and MP were 1.111%, 0.431%, 0.201%, 0.29% and 0.006% respectively. Present study result shows a higher Prevalence of TTIs than Tehran, Iran (0.515%)[21] and studies from India; Gujarat (0.77%)[22], Telangana (0.96%) [23], Ranchi (1.59%)[24], Odisha (1.89%) [25], central India(1.43%)[26] and Ahmedabad (0.58%) [27]. Compared to present study higher prevalence of TTIs reported from western region of Saudi Arabia (7.93%), Equatorial New Guinea (18.7%), Mozambique (37.39%) and Burkina Faso (24%)[20], Peshawar, Pakistan (5.33%)[28], WR Saudi Arabia(7.93%)[20], NW, Ethiopia (5.43%)[29], Eastern Ethiopia (7.06%)[30] and Brazil (Brazil%)[31]. Similar to our results, Most of the studies show higher prevalence of HBV among their blood donors [20-31].

Present study results are comparable with the published report (Table 5) on assessment of blood bank in India 2016 [32]. According to this repost in India overall prevalence of TTIs was 1.58% and prevalence of HBV,HCV,HIV and syphilis was 0.87%, 0.34%, 0.14% , 0.17% respectively which is lower than present study results and only MP (0.06%) was higher. According to this report, TTIs among donors of Delhi was 2.03% where as present study shows slightly higher prevalence of TTIs 2.038%. This is due to slightly higher prevalence of HBV, HIV and syphilis in present study compared to this report, however decreased prevalence of HCV and MP was observed. According to this report, highest prevalence of TTIs among Indian states (Table 5) was Puducherry (3.13%) and lowest was Kerala (0.56%). The highest prevalence of HBV, HCV, HIV, syphilis and MP was reported in Puducherry (2.12%), Panjab (1.35%), Puducherry (0.37%), Arunachal Pradesh (0.97%) and Andaman and Nicobar (0.76%) respectively. Most of the Indian states have higher prevalence of HBV among TTIs where as some states such as Chandigarh, Panjab, Manipur and Mizoram has higher prevalence of HCV and the state Arunachal Pradesh has higher prevalence of syphilis. Our result shows a lower prevalence of TTIs compared to some states of India such as Puducherry, Panjab, Mizoram, Madhya Pradesh, Arunachal Pradesh, Dadra and Nagar Haveli, Meghalaya and West Bengal [32].

The lower prevalence estimate of TTIs in the current investigation could be due to several factors, including the relatively low prevalence of blood-borne pathogens among the study population. This is due to public awareness, strict adherence to implementation of guidelines and SOP such as pre-donation counseling and medical examination to screen out high risk donors and sensitive screening tests, thus preventing the spread of infections. HBV is the most common kind of TTI evaluated in our study. The trend of screened HBV positive donors has variation, but shows a decreasing trend over the years, despite the fact that great progress has been done in India during the past thirty years to reduce HBV prevalence, it remains a critical concern. HBV in the community can be reduced by community-based awareness campaigns, which should focus on the chronic nature of the disease and its transmission. They should also stress the value of hepatitis B vaccine for the general population [23].

Some study report shows, the risk of TTI infection has no significant association with ABO and Rh D blood group [22, 25, 29]. Some study shows, donors with blood group O were highly contaminated with TTIs [19] while some study shows, O blood group had no association with TTIs [28]. Some study shows, risk of developing TTIs is more in O Rh(D)+Ve blood group donors and lowest in AB Rh(D)-Ve [20]. The author Shah RJ et al. has reported that syphilis has association with Rh Positive and Rh-negative blood group [27]. The author Mohammadali F et al. has reported, ABO and Rh blood groups not significantly associated with syphilis infection [21]. Present study results shows, syphilis infection has association with ABO blood group antigen. Significantly higher prevalence of syphilis was observed in donors having blood group O (no A and B antigen) and lower prevalence in donors having A and B antigen.

The author Zahra Naseri et al. has reported ABO blood groups not associated with HBV or HCV infection. They had also reported that, people having Rh negative blood group have less chance than others to have HBV [33]. The author Shah RJ et al. has reported that, HBV has association with Rh Positive and Rh-negative blood group [27]. The author Zufishan Batool had reported, blood group A has association with HIV and HBV infection. They had also reported that blood group O may have some protective influence against TTIs [28]. The author Mohammadali F et al. has reported, significantly higher percentage of HIV Ag/Ab in donor having blood group A and lower percentage of HBs Ag in donor having blood group O [21]. Present study shows HBV and HIV infection has no significant association with ABO and Rh (D) blood group antigen.

The author Sumit Bharadva et al. has reported, that blood group A negative has association with HCV infection [22] while other has reported, ABO and Rh blood groups has no associated with HCV infection [21]. The author Zahra Naseri et al. has reported HCV has no association with ABO blood groups and people having Rh positive have more chance of hepatitis C infection [33]. The author Behal R et al. found marginally high HCV seroprevalence in the Rhesus-positive compared to the Rhesus-negative group. They also fond significantly lower proportion of HCV prevalence in AB blood group compared to A, B, and O blood groups [34]. The author Xu Li et al. had reported, non-O blood types has a greater risk of HCV related hepatocellular carcinoma (HCC) than the patients having O blood group [35]. The author Najdat Shukur Mahmood has reported inβ- thalassaemia, patients HVC infection has significant association with blood groups A and B whereas patients having blood group O seemed to be protected or less susceptible to HCV infection [36]. The author Shah RJ et al. has reported that, blood group B positive had maximum sero-reactivity and O positive had minimum [27]. Our study results shows, a significant association between HCV and ABO blood group antigen. A significantly higher prevalence of HCV was observed in donors having blood group antigen B and lower in donors having A and O blood group antigen.

Although there was a noticeably lower rate of seropositive TTIs in the population under study, thorough knowledge of blood donors for TTIs is still required in order to further lower patient mortality and morbidity. Additionally, it makes room for all risk factors connected to TTIs. In order to better understand the clinical relationship between antigen receptors and infection, including their pathogenesis and association with blood group antigens, more research is required.

## Conclusion:

A low prevalence of TTIs positivity was seen among blood donors. Public awareness, proper counseling, medical examination and testing can help to minimize TTIs. Our study results shows ABO blood group has a association with HCV and VDRL infection.

## Authors' contributions:

The research design was created by Sanjay Kumar Thakur, Sompal Singh, Dinesh Kumar Negi and Anil Kumar Sinha. Sanjay Kumar Thakur conducted the literature search, data gathering, analysis, and manuscript preparation. All authors contributed equally to the contributed in data processing, interpretation, paper writing and critical revision of the final version of the text.

## Funding:

This study received no particular grants from funding sources in the governmental, commercial, or non-profit sectors.

## Figures and Tables

**Table 1:** Blood group wise and year wise frequency of TTIs in year 2020, 2021and 2022.

**year**		**A**	**B**	**O**	**AB**	**RHP**	**RHN**	**Total**
2020	Donor	1171(22.89)	1961(38.32)	1471(28.75)	514(10.04)	4828(94.35)	289(5.648)	5117(100)
	HBV	9(0.176)	21(0.41)	22(0.43)	5(0.098)	56(1.094)	1(0.02)	57(1.114)
	HCV	4(0.078)	15(0.293)	6(0.117)	1(0.02)	24(0.469)	2(0.039)	26(0.508)
	HIV-I/II	3(0.059)	6 (0.117)	2(0.039)	1(0.02)	12(0.235)	0(0)	12(0.235)
	VDRL	2(0.039)	9(0.176)	6(0.117)	3(0.059)	19(0.371)	1(0.02)	20(0.391)
	MP	0(0)	0(0)	1(0.02)	0(0)	1(0.02)	0(0)	1(0.02)
	Total TTIs	18(0.352)	51(0.997)	37(0.723)	10(0.195)	112(2.189)	4(0.078)	116(2.267)
2021	Donor	1791(23.2)	2899(37.55)	2284(29.59)	746(9.663)	7335(95.01)	385(4.987)	7720(100)
	HBV	20(0.259)	42(0.544)	19(0.2460	8(0.104)	84(1.088)	5(0.065)	89(1.153)
	HCV	5(0.065)	19(0.246)	5(0.065)	7(0.091)	32(0.415)	2(0.026)	36(0.466)
	HIV-I/II	3(0.039)	7(0.091)	5(0.065)	1(0.013)	15(0.194)	1(0.013)	16(0.207)
	VDRL	7(0.091)	5(0.065)	10(0.13)	0(0)	21(0.272)	1(0.013	22(0.285)
	MP	0(0)	0(0)	0(0)	0(0)	0(0)	0(0)	0(0)
	Total TTIs	35(0.453)	73(0.946)	39(0.505)	16(0.207)	152(1.969)	9(0.117)	163(2.111)
2022	Donor	945(23.12)	1517(37.11)	1199(29.33)	427(10.45)	3914(95.74)	174(4.256)	4088(100)
	HBV	15(0.367)	13(0.318)	10(0.245)	4(0.098)	40(0.978)	2(0.049)	42(1.027)
	HCV	3(0.073)	5(0.122)	2(0.049)	1(0.024)	11(0.269)	0(0)	11(0.269)
	HIV-I/II	0(0)	1(0.024)	5(0.122)	0(0)	6(0.147)	0(0)	6(0.147)
	VDRL	1(0.025)	2(0.049)	4(0.098)	0(0)	7(0.171)	0(0)	7(0.171)
	MP	0(0)	0(0)	0(0)	0(0)	0(0)	0(0)	0(0)
	Total TTIs	19(0.465)	21(0.514)	21(0.514)	5(0.122)	64(1.566)	2(0.049)	66(1.614)
Total	Donor	3907(23.08)	6377(37.678)	4954(29.27)	1687(9.968)	16077(94.99)	848(5.01)	16925(100)
	HBV	44(0.26)	76(0.449)	51(0.301)	17(0.1)	180(1.064)	8(0.047)	188(1.111)
	HCV	12(0.071)	39(0.23)	13(0.077)	9(0.053)	67(0.396)	4(0.024)	73(0.431)
	HIV-I/II	6(0.035)	14(0.083)	12(0.071)	2(0.012)	33(0.195)	1(0.006)	34(0.201)
	VDRL	10(0.059)	16(0.095)	20(0.118)	3(0.018)	47(0.278)	2(0.012)	49(0.29)
	MP	0(0)	0(0)	1(0.006)	0(0)	1(0.006)	0(0)	1(0.006)
	Total TTI	72(0.425)	145(0.857)	97(0.573)	31(0.183)	328(1.938)	15(0.089)	345(2.038)

**Table 2:** Trend of TTIs between year 2020, 2021 and 2022

**year**	**Donor**	**HBV (%)**	**HCV (%)**	**HIV-I/II (%)**	**VDRL (%)**	**MP (%)**	**T.TTI (%)**
2020	5117	57 (1.114)	26 (0.508)	12 (0.235	20 (0.391)	1(0.02)	116 (2.267)
2021	7720	89 (1.153)	36 (0.466	16 (0.207)	22 (0.285)	0(0)	163 (2.112)
2022	4088	42 (1.027)	11 (0.269)	6 (0.147)	7 (0.171)	0(0)	66 (1.615)
TOTAL	16925	188 (1.111)	73(0.431)	34(0.201)	49(0.29)	1 (0.006)	345(2.038)

**Table 3:** Blood group wise number of blood donors with TTIs negative and positive cases with their frequency in percentage (%) within the group

**Blood Group**	**Donor(N=16925)**	**HBV**		**HCV**		**HIV-I/II**		**VDRL**	
		**Negative(N= 16834)**	**Positive (N=191)**	**Negative (N=16951)**	**Positive (N=74)**	**Negative (N=16991)**	**Positive (N=34)**	**Negative (N=16976)**	**Positive (N=49)**
A	3907	3863	44	3895	12	3901	6	3897	10
	-23.08	-22.95	-23.04	-22.98	-16.22	-22.96	-17.65	-22.96	-20.41
B	6377	6300	77	6340	37	6363	14	6361	16
	-37.68	-37.42	-40.31	-37.4	-50	-37.45	-41.18	-37.47	-32.65
O	4954	4901	53	4938	16	4942	12	4933	21
	-29.27	-29.11	-27.75	-29.13	-21.62	-29.09	-35.29	-29.06	-42.86
AB	1687	1670	17	1678	9	1685	2	1685	2
	-9.97	-9.92	-8.9	-9.9	-12.16	-9.92	-5.88	-9.93	-4.08
A+AB	5594	5533	61	5573	21	5586	8	5582	12
	-33.05	-32.87	-31.94	-32.88	-28.38	-32.88	-23.53	-32.88	-24.49
B+AB	8064	7970	94	8018	46	8048	16	8046	18
	-47.65	-47.34	-49.21	-47.3	-62.16	-47.37	-47.06	-47.4	-36.73
A+B+AB	11971	11833	138	11913	58	11949	22	11943	28
	-70.73	-70.29	-72.25	-70.28	-78.38	-70.33	-64.71	-70.35	-57.14
A+B	10284	10163	121	10235	49	10264	20	10258	26
	-60.76	-60.37	-63.35	-60.38	-66.22	-60.41	-58.82	-60.43	-53.06
B+O	11331	11201	130	11278	53	11305	26	11294	37
	-66.948	-66.538	-68.063	-66.533	-71.62	-66.54	-76.47	-66.53	-75.51
A+O	8861	8764	97	8833	28	8843	18	8830	31
	-52.35	-52.06	-50.79	-52.11	-37.84	-52.05	-52.94	-52.01	-63.27
Rh(D) +Ve	16077	15896	181	16010	67	16044	33	16030	47
	-94.99	-94.43	-94.76	-94.45	-90.54	-94.43	-97.06	-94.43	-95.92
Rh(D)-Ve	848	838	10	841	7	847	1	846	2
	-5.01	-4.98	-5.24	-4.96	-9.46	-4.98	-2.94	-4.98	-4.08

**Table 4:** Association of TTIs with different blood groups (χ²; p-value, between negative and positive cases)

**Blood Group**	**DF**	**HBV**		**HCV**		**HIV-I/II**		**VDRL**	
		**χ²**	**p-value**	**χ²**	**p-value**	**χ²**	**p-value**	**χ²**	**p-value**
A,B,O, AB	3	0.73	0.866	6.358	0.095	1.54	0.673	5.289	0.152
(A+B+AB),O	1	0.216	0.642	2.1	0.147	0.597	0.44	4.382	0.036
Rh(D)+Ve, Rh(D)-Ve	1	0.193	0.66	3.091	0.079	0.306	0.58	0.089	0.765
A+O, B+AB	1	0.191	0.662	6.279	0.012	0.005	0.945	2.345	0.126
B+O,A+AB	1	0.108	0.742	2.592	0.107	1.396	0.237	1.628	0.202

**Table 5:** Prevalence (in percentage) of TTIs in Indian states according to Assessment of Blood Banks in India 2016 [32]

**States of India**	**HBV**	**HCV**	**HIV**	**Syphilis**	**MP**	**TTIs**
Puducherry	2.12	0.55	0.37	0.09	0	3.13
Panjab	0.65	1.35	0.14	0.49	0.01	2.64
Mizoram	0.94	1.24	0.3	0	0	2.48
Madhya Pradesh	1.14	0.1	0.08	0.36	0.56	2.24
Arunachal Pradesh	0.74	0.08	0.04	0.97	0.36	2.19
Dadra and Nagar Haveli	1.79	0.03	0.08	0.28	0	2.18
Meghalaya	0.78	0.47	0.16	0.73	0.04	2.18
West Bengal	0.9	0.52	0.26	0.35	0.02	2.05
Delhi	1.06	0.54	0.2	0.22	0.01	2.03
Andaman and Nicobar	0.85	0.27	0	0.12	0.76	2
Haryana	0.87	0.8	0.12	0.16	0.02	1.97
Andhra Pradesh	1.39	0.23	0.18	0.07	0.04	1.91
Bihar	1.42	0.14	0.16	0.05	0.07	1.84
Uttarakhand	0.76	0.67	0.1	0.13	0.13	1.79
Rajasthan	1.21	0.12	0.09	0.31	0.02	1.75
Uttar Pradesh	0.9	0.49	0.1	0.17	0.04	1.7
Maharastra	1.02	0.31	0.21	0.06	0.06	1.66
Manipur	0.59	0.83	0.15	0.04	0.01	1.62
India	0.87	0.34	0.14	0.17	0.06	1.58
Tripura	1.25	0.08	0.08	0.08	0.01	1.5
Karnataka	0.94	0.22	0.13	0.07	0	1.36
Chhattisgarh	0.68	0.17	0.13	0.3	0.04	1.32
Telangana	0.67	0.24	0.14	0.04	0.22	1.31
Odisha	0.8	0.17	0.11	0.13	0.08	1.29
Assam	0.54	0.24	0.12	0.3	0.03	1.23
Chandigarh	0.52	0.56	0.06	0.07	0	1.21
Sikkim,	0.59	0.26	0.06	0.19	0	1.1
Gujarat	0.59	0.13	0.1	0.2	0.01	1.03
Nagaland	0.34	0.27	0.26	0.14	0	1.01
Jharkhand	0.61	0.1	0.08	0.11	0.08	0.98
Tamil Nadu	0.68	0.11	0.05	0.07	0.01	0.92
Jammu and Kashmir	0.32	0.26	0.04	0.23	0.01	0.86
Himachal Pradesh	0.38	0.1	0.03	0.17	0.01	0.69
Goa	0.44	0.13	0.1	0.01	0.01	0.69
Daman and Diu		0.35	0.12	0.06	0.06	0.59
Kerala	0.28	0.17	0.05	0.04	0.02	0.56
